# Targeting vaccination against novel infections: risk, age and spatial structure for pandemic influenza in Great Britain

**DOI:** 10.1098/rsif.2010.0474

**Published:** 2010-10-13

**Authors:** Matt J. Keeling, Peter J. White

**Affiliations:** 1 Mathematics Institute and School of Life Sciences, University of Warwick, Coventry CV4 7AL, UK; 2 Modelling and Economics Unit, Health Protection Agency Centre for Infections, 61 Colindale Avenue, London NW9 5EQ, UK; 3 MRC Centre for Outbreak Analysis and Modelling, Department of Infectious Disease Epidemiology, Imperial College Faculty of Medicine, Norfolk Place, London W2 1PG, UK

**Keywords:** vaccination, targeting, novel infections, influenza, age structure, spatial structure

## Abstract

The emergence of a novel strain of H1N1 influenza virus in Mexico in 2009, and its subsequent worldwide spread, has focused attention to the question of optimal deployment of mass vaccination campaigns. Here, we use three relatively simple models to address three issues of primary concern in the targeting of any vaccine. The advantages of such simple models are that the underlying assumptions and effects of individual parameters are relatively clear, and the impact of uncertainty in the parametrization can be readily assessed in the early stages of an outbreak. In particular, we examine whether targeting risk-groups, age-groups or spatial regions could be optimal in terms of reducing the predicted number of cases or severe effects; and how these targeted strategies vary as the epidemic progresses. We examine the conditions under which it is optimal to initially target vaccination towards those individuals within the population who are most at risk of severe effects of infection. Using age-structured mixing matrices, we show that targeting vaccination towards the more epidemiologically important age groups (5–14 year olds and then 15–24 year olds) leads to the greatest reduction in the epidemic growth and hence reduces the total number of cases. Finally, we consider how spatially targeting the vaccine towards regions of country worst affected could provide an advantage. We discuss how all three of these priorities change as both the speed at which vaccination can be deployed and the start of the vaccination programme is varied.

## 1. Introduction

Vaccination has long been viewed as a vital tool in the armoury against infectious diseases. However, this perspective is largely based on our experience with endemic infections ([1,2], where a routine policy of vaccination can be used to increase the level of herd immunity [3]) and hence reduce or even eliminate the infection [4]. Examples of such approaches abound, from the eradication of smallpox and the virtual eradication of polio, to the long-running campaigns against childhood diseases such as measles, mumps and rubella [5,6], to the recently introduced schemes such as vaccination against human papillomavirus [7]. However, the recent experience with novel infections, such as SARS in 2003 and H1N1 pandemic influenza in 2009, have illustrated how vaccines are not a panacea due to the time needed to develop, manufacture and deploy the vaccine [8]. The UK, for example, had contracts to provide up to 132 million doses in the case of an influenza pandemic being declared, and 90 million doses were ordered in May 2009; however immunization did not begin until October. In total, only around five million doses [9] (approx. 400 000 to healthcare workers, about 37% of the 11 million people deemed in risk groups and about 20% of the three million children between 6 months and 5 years [10]) had been administered to people in priority groups by the end of February 2010, when the pandemic had effectively died out; therefore in February and April 2010 the orders were substantially reduced. Such statistics mean that we must carefully assess whether mass or targeted vaccination has a role in controlling future pandemics or large-scale outbreaks, and develop a range of robust models that can rapidly assess the benefits of vaccination and the best ways by which it can be targeted [8,11–15].

Here, we extend a relatively simple model for vaccination (of previously unvaccinated individuals) at a constant rate in three main directions to consider how different forms of heterogeneity can impact optimal vaccination. In particular, we focus on the trade-off between vaccinating those at risk of severe complications (if they become infected) compared with vaccinating individuals who are more epidemiologically active; we consider who this epidemiologically active set are in terms of age groups within the population; and we consider whether vaccination should be deployed randomly or if there are benefits from geographical targeting. Throughout, our aim is to develop relatively simple models, where assumptions and parameters are transparent, and where it is feasible to rapidly perform large sweeps over parameter space. Therefore, while all results are formulated based on the UK experience of the 2009 H1N1 pandemic (assuming *R*_0_ = 1.4 and a doubling time of around one week [16]), the model structure is sufficiently flexible that the qualitative results could pertain to a range of novel infections. (We believe that the methodology and results outlined here are likely to hold for any rapidly transmitted infection, with a short infected period and life-long immunity, and where a vaccine can be rapidly developed and manufactured.) Obviously, once a new infectious disease is identified, specialist models are required that can accurately capture the known dynamics and can incorporate the appropriate economic and logistical facets [8]. Producing accurate results from all types of model (including the very complex and relatively simple) relies on the availability of high-quality surveillance and detailed case records. In the initial stages of an epidemic, before these data are available, policy-makers need to know what range of scenarios are consistent with the initial data. Additionally, for many parts of the world, detailed data are never available. For simple models with fewer basic parameters, a more comprehensive sweep of parameter space is feasible allowing the rapid assessment of various targeting vaccination strategies for ranges of parameters that are broadly consistent with early qualitative information. We therefore believe that the results developed here provide generic insights into the optimization of vaccine deployment during a novel outbreak of a directly transmitted pathogen with lifelong immunity.

## 2. Basic model of vaccination

We first introduce the most basic model of vaccination in response to an infection that conforms to the simple SIR-type (susceptible–infectious–recovered) paradigm ([17–19]); this model will form the basic template for all the work that follows. We assume that the uncontrolled epidemic obeys the simple SIR model dynamics, such that susceptible individuals (of which there are *S*) can become infected and infectious by interaction with infected individuals (of which there are *I*), infected individuals recover at a constant rate and enter the recovered class (of which there are *R*) after which they are assumed immune for life. (We stress that all results presented are qualitatively invariant to the precise model formulation, in particular using a model with gamma-distributed exposed and infectious classes more reminiscent of the 2009 H1N1 pandemic [16].) In all the models that follow, we assume frequency-dependent mixing (such that the number of epidemiologically relevant contacts is independent of population size) and ignore the demographics of birth and death [17]—a reasonable assumption given the rapid time-frame of an epidemic. To this simple model, we add vaccination at a constant rate, *v*; we assume that individuals are vaccinated independently of their disease status but individuals are only vaccinated once, vaccination begins at time *T*, and a proportion *p* of vaccinated individuals are successfully immunized.2.1
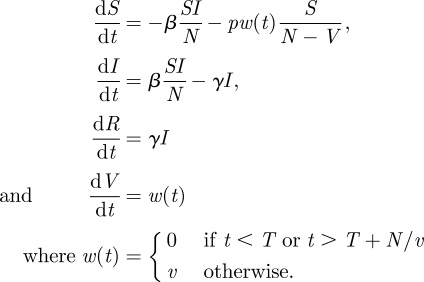
where *N* is the total population size. The precise way in which vaccination is implemented within the model ensures that a fixed number of individuals (*v*) are vaccinated per day, of which a fraction *p* are completely protected, and assumes that each person only receives one course of vaccine. If multiple doses of vaccine are required, or if protection only develops some time after vaccination, these can be included by delaying the time, *T*, at which immunization begins to take effect.

**Figure 1.**
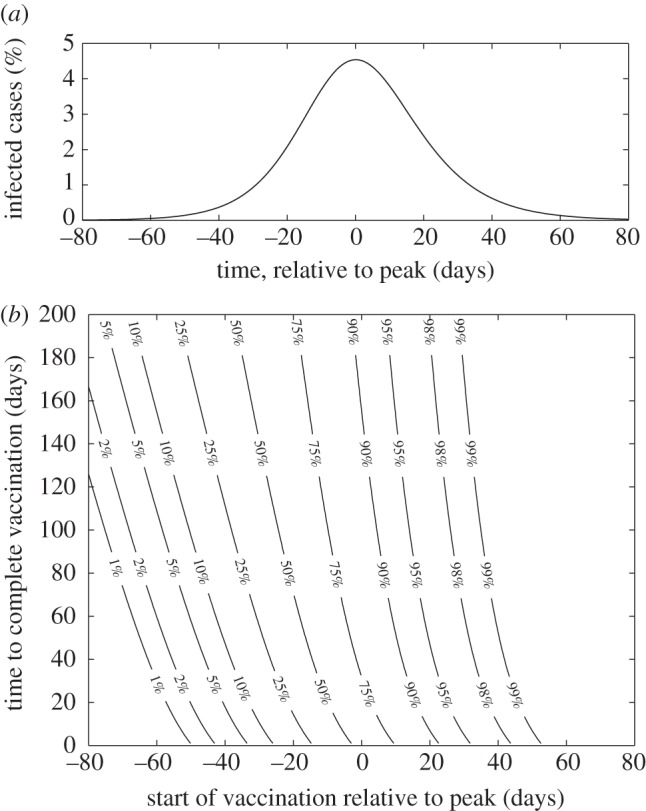
Results from the simple single homogeneous population model for vaccination (equation (2.1)). (*a*) Number of infected cases, with the time plotted relative to the peak incidence. (*b*) Contours of total number of cases (relatively to the unvaccinated maximum) when vaccination is begun at different times, and when the time to complete vaccination of the population varies between 1 and 200 days. (We set *R*_0_ = 1.4 and 1/*γ* = 4 days, to match the known epidemiological behaviour for pandemic influenza in England in 2009 [16].)

**Figure 2.**
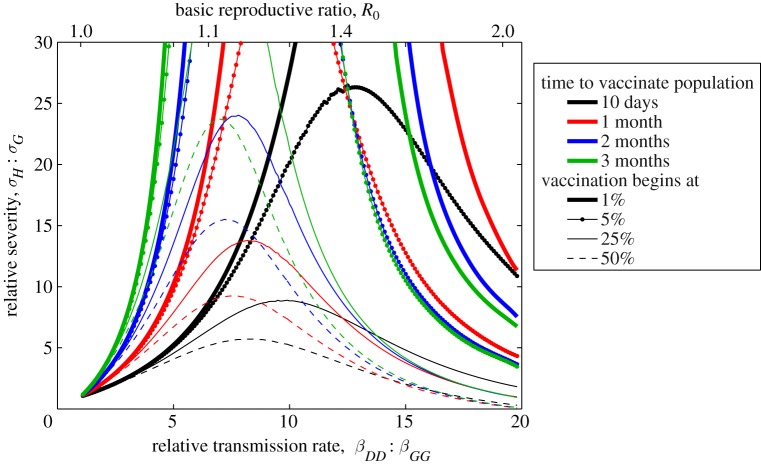
Epidemiological trade-off between initially vaccinating the group of dominant transmitters compared with initial vaccination of the group most likely to suffer severe consequences of infection. The curves are the contours where the two targeting strategies are equal (have the same total consequences integrated over the epidemic and summed over the groups); parameters above the curve favour vaccination of the severe-risk group first. The different coloured lines correspond to different speeds of vaccination (taking 10 days, 1, 2 or 3 months to vaccinate the entire population); the different line styles correspond to different points at which the vaccination campaign could begin (when the total number of cases have reached 1, 5, 25 or 50% of the unvaccinated total). (Throughout, we set all the transmission rates equal to *γ* except *β*_*DD*_ > *γ*; 1/*γ* = 4 days.)

Even this simplest of models confirms two simple rules-of-thumb regarding successful vaccination campaigns that seek to minimize the number of cases (figure 1). Firstly, that vaccination should begin as early as possible, so that susceptibles are depleted by vaccination before many cases arise; and secondly, that vaccination should be performed as rapidly as possible—both of which have been discussed before for a range of control measures [20,21]. From figure 1 it is also clear that an early start to a vaccination campaign is far more beneficial than faster vaccination of the population. It should be stressed that when considering an ongoing epidemic, the critical vaccination threshold for the elimination of an endemic infection or prevention of epidemic invasion (=1 − 1/*R*_0_) [17] no longer plays such a clear role. Instead, the primary aim should be to immunize many people in as short a time as possible, subject to trade-offs from economic costs or adverse effects of vaccination (such as that observed for smallpox [12,20]). Here, and in all the figures that follow, we have considered a wide range of vaccination speeds (*y*-axis in figure 1, line colours in figure 2, *x*-axes in figure 4); while some of these are extremely rapid and may be practically unachievable, the associated results are shown to provide a clearer picture of the most optimistic control scenario.

**Figure 3.**
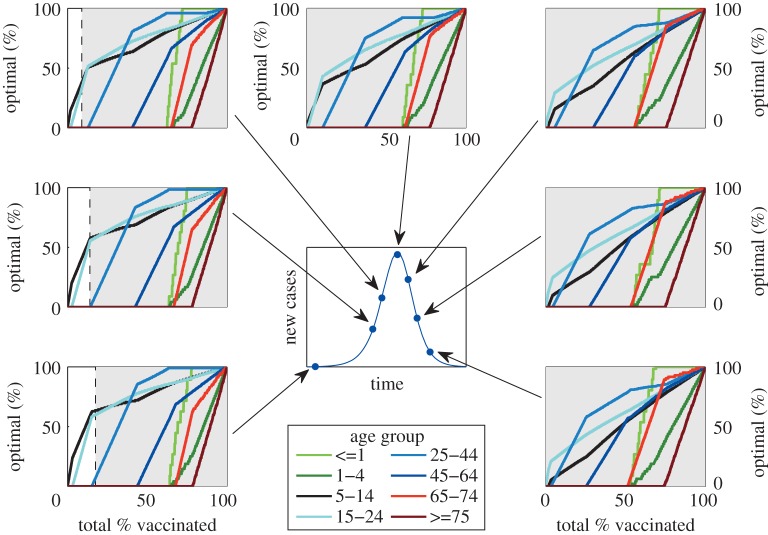
The sequential deployment of vaccination that for each dose targets the age class that offers the greatest reduction in the epidemic growth rate. As such, at each point in the vaccinate delivery schedule that the distribution is optimal in terms of minimizing the rate at which new cases are produced. The surrounding seven sub-graphs show this deployment of vaccine at different points during the epidemic (as indicated on the central epidemic curve); the shaded grey areas represent the vaccine levels where the reproductive ratio (*R*) is less than one, and therefore the epidemic is in decline. The population is broken into eight age groups, mirroring those used by the Health Protection Agency to report age-structured case reports.

**Figure 4.**
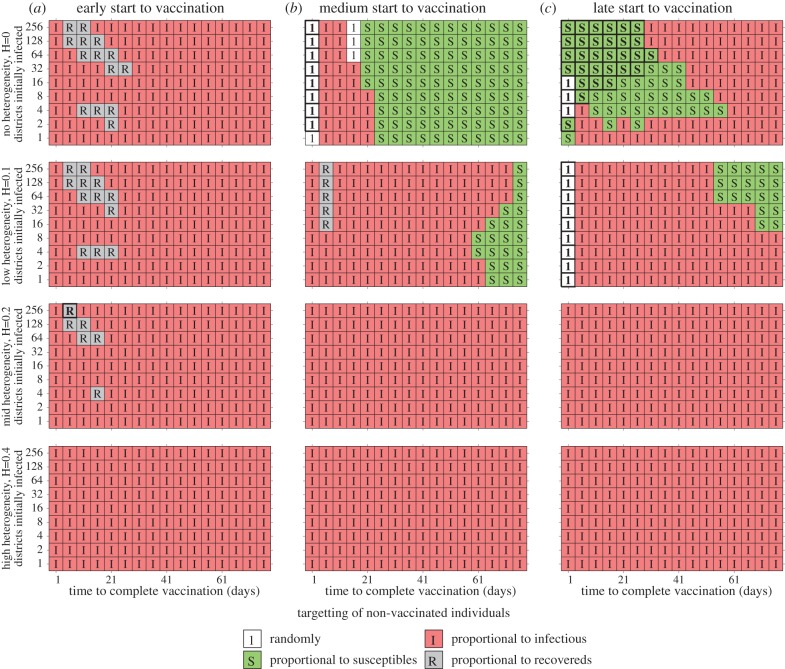
Results of spatially targeting vaccination in a deterministic metapopulation models of the 408 districts of Great Britain, linked by commuter movements from the 2001 census. Five simple alternatives for targeting unvaccinated individuals are considered, either randomly (shown as ‘1’ and white), or proportional to the proportion of susceptible (‘S’ and green), infected (‘I’ and red), recovered (‘R’ and grey) or vaccinated individuals (which is never optimal) in the district. For each parameter combination (of which 1926 are shown), 100 replicates were performed; for each replicate the transmission rate within each district and the distribution of cases is chosen randomly (within the prescribed values) and simulations were performed with all five alternative strategies. The letter (and colour) in each square corresponds to the strategy that generates the minimum number of cases the majority of times in the 100 replicates; letters in bold (with bold boxes) correspond to simple strategies that significantly outperform vaccination based on the proportion of infected. Rows correspond to different levels of heterogeneity in transmission, *H* = 0, 0.1, 0.2, 0.4; columns correspond to when the vaccination campaign starts, defined in terms of the number of recovered individuals, (*a*) early vaccination (begun when *R* = 100, 100 recovered cases nationally), (*b*) vaccination begins when *R* = 0.1N, and (*c*) vaccination starts late (begun when *R* = 0.2N). (*R*_0_ = 1.4, 1/*γ* = 4 days, *β*_*d*_ = *R*_0_*γ* exp(*Hξ*_*d*_); where *ξ*_*d*_ is a random variable, normally distributed with mean 0 and a variance of 1.)

Given this absence of clear epidemiological trade-offs in this simple model, we need to consider a range of more structured models in which we can consider the trade-offs involved with the prioritization of different groups for vaccination.

## 3. Who to vaccinate: high health risk or high transmission groups?

When targeting a vaccination campaign (especially against the 2009 H1N1 influenza strain), there are often two competing priorities: minimization of transmission by immunizing those individuals that are most epidemiologically important; and minimization of the effects of the disease by immunizing those individuals that have the most severe health consequences when infected. (We note that for other infections these two groups may strongly overlap, in which case there is no conflict of priorities to resolve.) To tease apart these conflicting ideals, we extend the simple model above by having three groups [18,19,22,23]: the dominant transmitter group (denoted by a subscript *D*), the group at highest risk of severe health complications if they become infected (denoted by a subscript *H*) and the rest of the general population (denoted by a subscript *G*). These groups obey the basic equation (2.1) with two main modifications: firstly, the transmission dynamics are coupled through a ‘who acquires infection from whom’ matrix (*β*); and secondly, vaccination is prioritized so that either the dominant transmitter group (*D*) or the severe health risk group (*H*) is vaccinated first, followed by the other group (either *H* or *D*), finally followed by the general population (*G*), (see electronic supplementary material). Here we have ignored the possibility that there is a group that are both dominant transmitters and at high risk of complications—obviously if such a group exists then it should be prioritized for vaccination before all others.

Obviously, an epidemiological model with three interacting groups has a large number of associated parameters, making a comprehensive sweep of the entire parameter space impractical and difficult to visualize. Instead, we show results from a relatively restricted scenario, but comment that these results are representative of all plausible scenarios that have been considered. In particular, we constrain the number of individuals in the three groups to be *N*_*D*_ = *N*_*H*_ = 0.1 N and *H*_*G*_ = 0.8 N, and constrain the transmission rates between all groups, except within the epidemiologically important group, to be equal (*β*_*XY*_ = *γ*, except when *X* = *Y* = *D*). We note that different forms and parameters within this transmission matrix can potentially lead to different optimizations of vaccine [22].

We now consider the optimal prioritization (either group *D* first or group *H* first), as four key parameters are varied: the transmission rate within the dominant transmitter group, *β*_*DD*_; the relative adverse consequences of infection for the three groups, *σ*_*H*_ > *σ*_*D*_ = *σ*_*G*_; the timing for the start of the vaccination programme, *T*; and the speed with which the population is vaccinated, *v*. Here the consequences of infection could capture a variety of measures, from risk of symptoms if infected, to concepts such as loss of QALYs (auality-adjusted life years), to risk of hospitalization, to risk of mortality associated with infection. The curves shown in figure 2 separate regions of parameter space where one form of prioritization is optimal, in terms of minimizing the total consequences of infection over the entire epidemic and across all three groups; regions above and to the left of the curves are where it is best to initially target vaccination towards the group with potentially severe health complications.

Two clear conclusions can be drawn. More rapid vaccination (larger *v*) generally favours prioritizing vaccination towards the group with potential health consequences, as does a later onset of vaccination (larger *T*). However, it should be noted that for either extremely rapid or extremely slow vaccination (or extremely late start of the vaccination programme), the differences between the two prioritization schemes will be minimal. We can obtain some estimate of consequences by examining the figures released by the Chief Medical Officer for England [9]: of 342 confirmed deaths in England from H1N1 by the middle of March 2010, 52 per cent had severe underlying health problems while only 18 per cent were classified as previously healthy; similarly nearly 50 per cent of hospitalized patients were considered to have underlying conditions. These percentages, together with the fact that only 10 per cent of the general population are considered to have health problems, lead to estimates of *σ*_*H*_ : *σ*_*G*_ of atleast 9:1. Therefore, while there are a range of scenarios in which it would be optimal to target the dominant transmitters first, these tend to be in relatively extreme portions of parameter space, when the transmission rate *β*_*DD*_ is very high and vaccination begins very early in the epidemic; for the vast majority of realistic scenarios, it is generally optimal to target vaccination towards those members of the population with underlying health problems first, before tackling the dominant transmitters and the rest of the general population.

## 4. Who are the epidemiologically important group?

Analysis of the 2009 pandemic to date in Britain, and elsewhere, indicates that there are some strong age-dependent signatures. Most notably, school children have suffered the greatest per capita burden of infection as recorded by surveillance systems, whereas pre-school children have experienced the greatest per capita level of severe infection (as measured by hospital admissions), while the over 65 age group were most likely to suffer severe problems if they became infected [9,16]. These different age-dependent effects are due to several interacting and conflicting factors: the highly structured mixing between age groups, the age-related susceptibility to infection and the age-dependent risk of severe symptoms following infection. To combine these factors require a mathematical model based on the available age-structured information. Here we use data from the POLYMOD study [24] to parametrize age-related mixing patterns, where *P*_*a,b*_ captures the estimated contact rate between individuals of ages *a* and *b*. In the electronic supplementary material we also show that an age-dependent susceptibility vector (*q*_a_) can be estimated such that the early dynamics, as predicted by the dominant eigenvector of the transmission matrix, agree with the observed early age-structured distribution of infection. However, for greater generality, we set 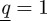 in figure 3, although even using the estimated age-dependent susceptibility from the 2009 pandemic, together with the impact of school holidays, does not dramatically change the predictions (see electronic supplementary material).

We use the age-dependent transmission matrix *β* to determine an optimal priority for a rapid age-dependent vaccination programme [23,25–27] (figure 3). The methodology is as follows: for each single dose of vaccine, we consider which age class should be immunized such that the resultant growth-rate (as predicted by the dominant eigenvalue) is minimized; repeating this process successively generates a vaccination strategy that should rapidly control the epidemic for any given level of vaccine coverage. (We note that [27] provide an alternative, more analytical method of minimizing the eigenvalue, which is equivalent to our approach once the total level of vaccine exceeds a threshold.) The vaccination strategies given in figure 3 therefore inform about the instantaneous epidemiological significance of each age group at a particular point during an epidemic. We do not claim that these strategies are truly optimal (in terms of minimizing the predicted total number of cases across all possible distributions of vaccine), nor that such strategies are entirely relevant if vaccination is slow relative to the epidemic timescales (owing to the changes in the priorities we observe as the epidemic progresses, as shown in the sub-graphs). However, these age-specific vaccination profiles do provide an intuitive means of sequentially and efficiently increasing the vaccination coverage at any given point in the epidemic and have been found to agree with the optimal distribution of a fixed quantity of vaccine that minimizes the dominant eigenvalue [27]. What is crucial to note in these plots is that they represent a theoretical ideal when vaccine supply is limited rather than an achievable goal. If vaccine is not in short supply then it is clearly always better (both in terms of reducing growth rate and total epidemic size) to vaccinate someone than not, even if this leads to substantial deviation away from the optimal age profile. Therefore, these plots inform about possible prioritization of the vaccine campaign.

In the early stages of the epidemic, before there has been significant depletion of susceptibles, the predicted vaccination strategy initially targets the 5–14 and 15–24 year old age groups; vaccination should then begin in the 25–44 age group, with older ages (greater than 45) and the younger ages (under 5) not being targeted until vaccine coverage exceeds 50 per cent. What is somewhat counterintuitive is that the optimal deployment of vaccine could be partial in many age classes. For example, if 50 per cent of the population can be vaccinated, then the optimal strategy would be to attain highest levels of coverage in the 25–44 year old age group and less in the ages 5–24, despite the fact that the 5–24 year old age groups are favoured for early vaccination before the 25–44 year old age group. A second feature emerges as vaccination is begun later in the epidemic. Because the epidemic process has already depleted much of the susceptible population in the most epidemiologically active age classes (namely the 5–14 year olds and 15–24 year olds), there is a decreased benefit from large-scale vaccination of these age groups. It should be stressed that both the distribution of optimal vaccination at a given time, and the way this changes as the epidemic progresses, are critically dependent on the number of individuals in each age group, the mixing matrix (*β*_*a,b*_) and hence the age-dependent susceptibility (*q*_a_). This means that precise details will depend on the detailed epidemiology of the infection under investigation, and are therefore likely to vary between different strains of influenza—for example, with models parametrized to match the 2009 pandemic in England (see electronic supplementary material), the 1–4 year old age group plays a far more dominant role.

## 5. Where should vaccination be targeted?

The final important question to address when delivering a vaccination programme, is whether there are any advantages in spatially targeting its deployment [28,29]. Obvious choices could be to target regions with a high immediate burden (targeting based on current proportion of infectious cases), or to target regions that are likely to have many cases in the future (targeting based on current proportion of susceptibles), or simply to vaccinate randomly with a fixed *per captia* rate. Determining the optimal spatial targeting of vaccination is difficult because the impact of vaccination is long-lasting, cumulative and nonlinear, meaning that a generic understanding cannot be generated by considering the impact of low vaccination levels, nor can the action of vaccination be considered piece-wise as it was above. The only viable option is to simulate the dynamics with a variety of strategies and ascertain which performs the best numerically, however the issue now becomes the number of possible ways in which the vaccine could be distributed.

We use a deterministic metapopulation model of the progress of an influenza-like infection in the 408 districts of Great Britain, linked by the commuter movements recorded in the 2001 census, and consider a wide range of epidemiological scenarios; with different onset times for the start of vaccination (*T*), different numbers of districts initially infected and different levels of transmission heterogeneity, as captured by different *β*_*d*_ in each district ( see electronic supplementary material). (Because of the added complexity of dealing with an explicitly spatial model, other forms of heterogeneity such as age or risk structure have been ignored, although their inclusion could be formulated in a similar manner to that described above. We have focused on modelling at the district scale as targeting could be practically achieved as such a resolution; finer scale targeting such as at the ward level would lead to a enhanced vaccination scheme but is unlikely to be practical.) The vaccination level in district *d* is given as a function of the current epidemiological conditions in that district:5.1
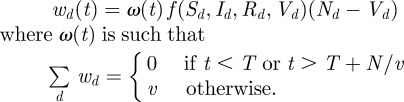


As with equation (2.1), this formulation ensures the vaccination of a constant number (*v*) of (previously unvaccinated) individuals each day, targeting unvaccinated individuals in each subpopulation at a *per capita* rate proportional to *f*. We have adopted the convention that variables and parameters with subscripts refer to specific districts, while those without subscripts refer to the entire population.

The initial assessment was to consider targeting (*f*) that was proportional to one of the standard model variables (*S*_*d*_/*N*_*d*_, *I*_*d*_/*N*_*d*_, *R*_*d*_/*N*_*d*_, *V*_*d*_/*N*_*d*_) or purely random (*f* = 1); results are shown in figure 4. (Alternative more complex methods of targeting are shown in the see electronic supplementary material.) Each square is colour-coded and labelled according to which targeting consistently generates the lowest number of cases (from 100 replicate simulations with random initial distribution of infection). When there is no regional heterogeneity in epidemiological parameters (*β*_*d*_ = *β*, top row), then the best strategy depends on the speed of vaccination (*x*-axis), the time when the vaccination campaign begins (column) and to a lesser extent the number of districts initially infected (*y*-axis). Early vaccination (begun as soon as cases are detected) generally favours targeting of vaccination proportional to the current proportion of infected cases (*f*_*d*_ = *I*_*d*_/*N*_*d*_); if vaccination takes more than three weeks to complete, then a later start to the vaccination programme (begun when 10% of the population have been infected and therefore approx. 20% of the way through the uncontrolled epidemic) favours targeting proportional to the proportion of remaining susceptibles in the population (*f*_*d*_ = *S*_*d*_/*N*_*d*_)—therefore, areas that have seen relatively less infection are favoured. Finally, if vaccination is implemented, even later (begun when 20% of the population have been infected and therefore approx. 40% of the way through the uncontrolled epidemic) targeting according to the proportion of susceptibles is again favoured when the epidemic is relatively dispersed and vaccination is relatively prompt.

The top row of figure 4 corresponds to the unlikely scenario when each spatial district has identical parameters; in reality, there is likely to be significant heterogeneity in transmission between different areas owing to social and demographic factors. In the 2009 influenza pandemic in the UK, it was observed that the epidemic grew more rapidly in London and the West Midlands, possibly owing to such a mixture of socio-demographic factors. Unfortunately, the degree of such heterogeneity is difficult to estimate and is likely to vary between outbreaks; we therefore force the transmission rate within each district to be log-normally distributed about the mean, with the variance of the distribution controlled by the spatial heterogeneity (*H*). (In particular, the transmission rate is given by *β*_*d*_ = *β* exp(*Hξ*_*d*_), where *ξ*_*d*_ is a random variable, normally distributed with mean zero and a variance of one.) When such heterogeneity is added, in the overwhelming majority of scenarios (and certainly when *H* ≥ 0.2), targeting in terms of the proportion of individuals currently infectious (*f*_*d*_ = *I*_*d*_/*N*_*d*_) is the best strategy. This advantage for targeting in terms of current infectious cases is due to two main reasons: firstly, when there is significant heterogeneity in growth rates it will naturally target vaccination at those regions likely to suffer larger epidemics; secondly, by controlling infection in the worst affected area, the spread of infection to other areas is reduced and therefore more time is gained to vaccinate the remaining areas. (As a further refinement to targeting in the electronic supplementary material, we consider *f* = (*I*_*d*_/*N*_*d*_)^*α*^ and seek the exponent, α*, that minimizes the predicted number of cases.)

## 6. Discussion

The mass use of an effective vaccine clearly has the potential to provide major health benefits in terms of a reduction in the total number of infected cases, and therefore a reduction in the total number of adverse effects [1,3,7]. However, when used against an ongoing epidemic, the logistical constraints in terms of the speed with which vaccine can be manufactured and administered means that its deployment must be carefully targeted [8,11–15,23,26,27]. Many of these have focused on particular aspects of targeted vaccination (such as age- or risk-based) or have developed approximate [26] or analytical [27] methods for optimal targeting. Here, using standard differential equation models augmented to reflect particular heterogeneities, we have attempted to provide a relatively general framework that will be familiar with public-health epidemiologists and other non-specialist modellers. In general, our results agree with those of earlier studies although we have often attempted to consider a far-wider parameter space, with the aim of spanning much of the parameter uncertainty that is likely to arise during the early stages of an epidemic.

Three forms of targeting have been considered, in terms of risk groups, age structure and spatial location, to derive general insights into the benefits of targeted vaccination. Throughout we have focussed on the development of highly parsimonious models, where assumptions and parameterization are relatively transparent; however, even for these models the parameter space is often too large to be visualized, in such cases model parameters are based on observations from the 2009 influenza pandemic in Great Britain. Despite this large parameter space, several generic conclusions can be drawn about the optimal deployment of vaccine:

— as predicted by even the simplest models, vaccination campaigns are most effective when they are applied rapidly and early in an epidemic before many cases arise. In fact, figure 1 shows that an early start to the vaccination campaign (and therefore rapid development of the vaccine) is of more advantage than administering the vaccine quickly;
— owing to the natural levels of heterogeneity displayed within the population, it is generally better to initially target vaccination towards those groups likely to have severe symptoms rather than those groups that are most responsible for transmission (figure 2). To some extent, this is due to the ease with which those groups that are likely to suffer complications can be identified. The parameter regime in which it is better to initially vaccinate the severe risk groups, is extended when the vaccination campaign begins later, or when the population can be vaccinated more rapidly;
— once the groups with severe health risks have been protected (or it has been deemed better to target other groups), attention naturally focuses on which elements of the population are the most epidemiologically active and the benefits associated from vaccinating these individuals. Two main results emerge (figure 3); firstly, any one group should not be targeted to the exclusion of all others, the best strategy is generally a mixed strategy. Therefore, while it is generally predicted (conditional upon age-dependent susceptibility) that school-age children should be the initial focus of vaccination, the optimal strategy does not concentrate on achieving complete coverage of this group, instead it is best to target other groups simultaneously;
— in addition, the best groups to target for vaccination vary as the epidemic progresses. Most notably, age groups that play the dominant epidemiological role are rapidly depleted and therefore their importance wanes as the epidemic progresses, and hence the advantages of targeted vaccination compared with random vaccination also decline; and
— although it is difficult (if not completely impractical) to calculate the true optimal spatially targeted vaccination policy, instigating at least some measure of targeting has significant advantages. Depending on the level of spatial heterogeneity in transmission within the population (which could reflect underlying demographic heterogeneity), targeting of vaccination towards regions that are currently experiencing high levels of infection generally reduces the total number of cases. The intuitive reason for this targeting is that by concentrating on centres of infections (and reducing the immediate growth rate), it buys extra time to vaccinate other regions. Achieving such a targeting in practice would require health services to be able to rapidly shift resources around the country, and only applies when there is a national limit to the deployment of vaccine. However, these results indicate two important points: firstly, that even when vaccination schemes are administered locally, there are likely to be strong advantages in spatially targeting any additional national resources; and secondly that the likely human reaction for there to be a greater demand for vaccine in regions with a higher proportion of cases would assist in control.

Because of the relatively simplistic nature of the models developed in this paper, several caveats should be made regarding the results. The first is that vaccination campaigns are unlikely to achieve a constant level of uptake over time; a more realistic assumption is that vaccination initially begins slowly due to logistical issues, builds to a plateau, but may finally decline if the epidemic begins to wane during the period of the vaccination programme and there is less incentive for individuals to be vaccinated. Associated with this is the fact that not all individuals are prepared to be vaccinated, and in particular parents in the UK are often anxious about vaccinating children; therefore, the optimal targeting of vaccination is unlikely to be possible and it may simply be better to vaccinate any individuals who wish to be vaccinated. The second issue, which applies to figures 2 and 3, is that it may not always be possible to identify or target relevant risk groups. For example, there may be considerable overlap between the age groups used in figure 3, and the groups at risk of severe symptoms defined in figure 2; this was undoubtedly the case in 2009 (as well as previous pandemics), where age was often a key contributing factor to both the risk of infection and the risk of complications. Throughout, we have assumed a perfect vaccine, which offers 100 per cent protection to all those vaccinated. In practice, this is never realized, all vaccines fail to some degree; however, there are two ways in which this failure can be modelled. The first is an all or nothing approach in which a fraction of all vaccinations fails to generate any protection, while the remaining offers full protection; in this case, the results of our model natural extrapolate based on considering the numbers protected. The second approachis to consider ‘leaky’ vaccines that offer partial protection reducing either susceptibility or onward transmission; given the relatively low reproductive ratios considered within this paper, we believe that our results are likely to generalize to this case. A further issue related to figure 2 is the ethics of vaccinating the epidemiologically important groups in order to protect the group with severe health risks. While for influenza the vaccine has little associated risk and therefore it may be argued that the health benefits to the epidemiologically important (but healthy) groups outweigh the dangers, the same may not be true for other pathogens and the associated vaccine. Additionally, we have generally used deterministic models and hence assumed that vaccine would be used to mitigate the impact of infection rather than to prevent an epidemic occurring. This is particularly pertinent to the metapopulation model (figure 4) when we neglect the possibility of using vaccine to eradicate infection in the early stages and prevent its spread to the remainder of the country as exemplified in Ferguson *et al.* [11]. We feel this is a reasonable assumption given that most novel infections will be seeded continually by imports from abroad, and vaccine is unlikely to be available at the start of the epidemic. Finally, throughout we have assumed that there is a strong public demand for vaccine. Clearly, demand will vary both with disease severity and public perception of the infection; for example, the relatively mild nature of the 2009 pandemic meant that demand and uptake of the vaccine was low. However, if the infection has severe health implications, and therefore there is a clear need to optimize control measures, then demand for vaccination is likely to be substantial.

The models in this paper, and therefore the results generated, are not designed to replace very detailed simulations parametrized to match epidemiological data from a given outbreak; however, they do provide a high degree of generality that is difficult to obtain with more case-specific simulations and hence provide a rapid assessment of conflicting methods of targeting vaccination. Often public-health action has to be taken in the absence of critical information: levels of prior immunity in the population affect the transmission patterns, but it takes time to develop the appropriate serological test; parameters such as case-fatality ratios or the probability of hospitalization are difficult to estimate in real time as an epidemic progresses; and although clinical trials can measure the theoretical vaccine efficacy, vaccine effectiveness in practice can only be determined once the vaccination programme has begun. Given this range of uncertainties, models can be best used to assist policy-makers by examining a range of scenarios ahead of time; so decisions can be taken based on the range of scenarios that are consistent with the available data at the time that the decision has to be taken. Simple models often allow us to partition parameter space into clearly defined regions where a particular strategy is optimal; and while precise parameters may be difficult to estimate early in an epidemic, there may be sufficient evidence to suggest that the infection parameters lie within one of these prescribed regions. As such, the simple models developed here provide useful policy guidance before or during the early stages of an epidemic before there are sufficient data to parametrize more detailed simulations.
